# Case Report: Chimeric Antigen Receptor T Cells Induced Late Severe Cytokine Release Syndrome

**DOI:** 10.3389/fonc.2022.893928

**Published:** 2022-06-01

**Authors:** Jinping He, Na Xu, Hongsheng Zhou, Ya Zhou, Di Wu, Ruochong Zhao, Tong Lin, Ju Xu, Rui Cao, Peng Li, Qifa Liu

**Affiliations:** ^1^ Department of Hematology, Nanfang Hospital, Southern Medical University, Guangzhou, China; ^2^ Center for Cell Regeneration and Biotherapy, Guangzhou Institutes of Biomedicine and Health, Chinese Academy of Sciences, Guangzhou, China; ^3^ Institute of Hematology, Medical College, Jinan University, Guangzhou, China; ^4^ Department of Pediatric Endocrinology and Genetic Metabolism, Guangzhou Women and Children’s Medical Center, Guangzhou Medical University, Guangzhou, China

**Keywords:** chimeric antigen receptor T cell (CART), severe cytokine release syndrome (sCRS), late period, re-expansion, haemophagocytic lymphohistiocytosis (HLH), cutaneous toxic effect

## Abstract

**Background:**

Severe cytokine release syndrome (sCRS) has emerged as an adverse complication in the early period of chimeric antigen receptor T cell (CART) therapy, while whether sCRS occurs in the late period remains unknown. Here, we reported two patients with late sCRS.

**Case Presentation:**

Case 1 was a 34-year-old female with refractory Philadelphia chromosome-positive B cell acute lymphoblastic leukemia. She achieved complete remission (CR) but experienced grade III CRS and hemophagocytic lymphohistiocytosis (HLH) 41 days after CD19-targeted CART (CART19) cells and CD22-targeted CART (CART22) cells infusion. Ineffective to tocilizumab and HLH-94 protocol (dexamethasone and etoposide), she died of a cerebral hemorrhage on day 55 after CART therapy. Case 2 was a 38-year-old male with IgG kappa multiple myeloma. He received autologous BCMA-targeted CART (BCMA-CART) therapy 4 months after HLA–matched sibling (sister) donor transplantation and developed grade III CRS 163 days after CART administration, characterized by fever, hypotension, and skin lesions. Effective to methylprednisolone and tocilizumab, his clinical response persisted for over 6.0 months.

**Conclusion:**

Severe CRS could occur in the late period after CART therapy as re-expansion of CART cells possessed the potential risk for late sCRS.

## Introduction

Chimeric Antigen Receptor T Cell (CART) therapy has dramatically expanded therapeutic options among those with high-risk B-cell malignancies (1, 2). Despite the high overall remission rate, early and late complications may cause significant morbidity and even mortality (3–6). As for the early complications, the most common toxicity after CART administration is cytokine release syndrome (CRS), which has been reported to be as high as 77%-100% in CART19 clinical trials (7–9). CRS onset time ranged from 1-22 days (the median onset time: 3 days) and the duration lasted for 1-36 days (the median duration: 8 days) (8, 9). Occurring in 10%-30% of patients with CRS, severe CRS (sCRS) is manifested by fever, hypotension, respiratory distress, nervous system symptoms, and end-organ dysfunction (4, 9). Serious adverse events after 30 days were rarely reported though CART in the blood could be observed as long as 23.1 months (8). As for the most common late complications, cytopenia that had not resolved by day 28 was reported in 53%-78% of patients (5, 8, 10). Additionally, less than 20% of them had prolonged cytopenia 3 months after administration (5). However, prolonged cytopenia did not become the leading cause of death in patients treated with CART (5, 8, 10). Here we report two patients who suffered from late sCRS at an atypical onset time. One developed into grade III CRS and carHLH on day 41 after CART infusion while the other experienced grade III CRS on day 163 after CART infusion. In both cases, elevated levels of CART and IL-6 could be detected in the peripheral blood. To the best of our knowledge, this is the first report regarding patients with CART inducing late sCRS.

## Case Description

### Case 1

**Figure 1:**
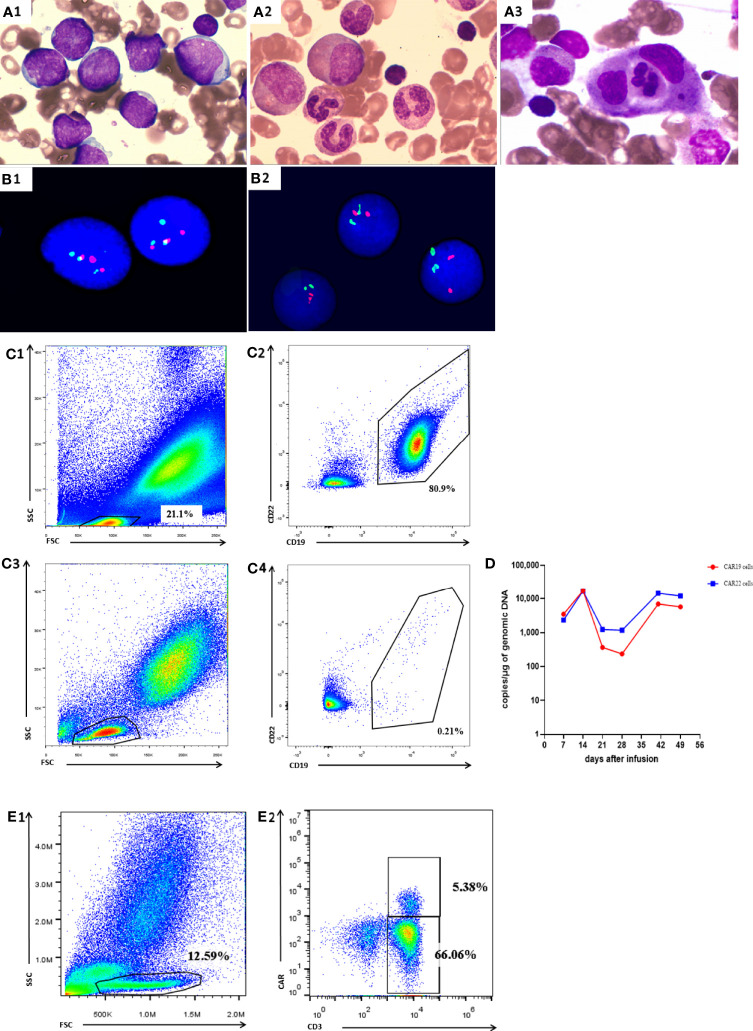
Manifestations in Case 1. **(A1)** BM smear before CART therapy showed that immature lymphocytes accounted for 92.1% total cells. **(A2)** BM smear on day 30 showed that immature lymphocytes accounted for 4.5% total cells. **(A3)** BM smear on day 41 showed that less than 0.5% immature lymphocytes and about 16% hemophagocytic cells in NEC. **(B1)** FISH before CART therapy revealed 81% BCR/ABL gene fusion. FISH on day 30 **(B2)**, **(B2)** FISH on day 30 after CART therapy revealed undetectable BCR/ABL gene fusion after CART therapy revealed undetectable BCR/ABL gene fusion. **(C1, C2)** BM FCM analysis before CART therapy exhibited 80.9% lymphocytes characterized by CD19+CD22+. **(C3, C4)** BM FCM analysis on day 30 exhibited 0.21% lymphocytes characterized by CD19+CD22+. **(D)** qPCR showed detectable CAR19 and CAR22 DNA in peripheral blood after CART therapy. **(E1, E2)** on day 41.

A female patient aged 34 years was diagnosed with Philadelphia chromosome-positive B cell acute lymphoblastic leukemia. After two courses of VDLP regimen (vincristine, idarubicin, L-asparagines, and prednisone) combined with dasatinib, she did not achieve complete remission (CR) as bone marrow (BM) smear showed that immature lymphocytes accounted for 28% of nonerythroid cells (NEC). The laboratory results before CART therapy were as follows: 1) white blood cell (WBC), hemoglobin (Hb), and platelet (Plt) were 2.74 × 10^9^/L, 77 g/L, 65×10^9^/L, respectively; 2) BM smear showed that immature lymphocytes accounted for 92% of NEC (**Figure 1A1**) and fluorescence *in situ* hybridization (FISH) showed 81% BCR/ABL fusion gene (**Figure 1B1**); 3) the flow cytometry (FCM) analysis exhibited that 80.9% of lymphocytes were characterized by CD19^+^CD22^+^ (**Figures 1C1**, **C2**). Based on these results, the patient was recruited into CART clinical trial for relapsed or refractory B-cell malignancies (ChiCTR-OPN-16008526). Patients were given fludarabine 25 mg/m^2^ and cyclophosphamide 300 mg/m^2^ on day -4, -3, and -2, followed by intravenously administration of 2.4 × 10^6^/kg CART19 and 2.1 × 10^6^/kg CART22 separately on day 0 (11). Adverse events were not observed during CART infusion. On day 30, she achieved hematologic CR (**Figure 1A2** and **Table 1**) and BCR/ABL fusion was undetectable (**Figure 1B2**) by FISH, 0.14% BCR/ABL mRNA was detected by qPCR, and 0.21% CD19^+^CD22^+^ lymphocytes were found by BM FCM (**Figures 1C3**, **C4**).

**Table 1:** The laboratory results in Case 1.

Date After CART	WBC (10^9^/L)(Ref: 4-10)	Hb (g/L)(Ref: 110-150)	Plt (10^9^/L)(Ref: 150-390)	ALT (U/L)(Ref: 5-40)	AST (U/L)(Ref: 8-40)	CRP (mg/L)(Ref: 0-5)	PCT (μ g/L)(Ref: 0-0.5)	PT (s)(Ref: 11-14)	APTT (s)(Ref: 25-37)	FIB (g/L)(Ref: 2-4)	D-Dimer (mg/L)(Ref: 0-0.55)	Ferritin (ng/ml)(Ref: 15-200)	IL-6 (pg/ml)(Ref: 0.37-0.46)
Day30	4.48	102	184	32	27	2.14	0.12	–	–	–	–	–	–
Day41	5.67	116	150	28	15	1.39	0.16	13.5	34.1	3.29	0.73	68.2	395.96
Day43	2.89	78	46	35	18	3.13	0.34	16.6	35.7	2.96	–	–	489.43
Day45	2.79	61	15	78	86	2.17	0.21	17.1	56.2	1.24	23.13	178,678.1	3366.65
Day47	2.34	53	4	–	–	–	–	18.2	47.2	0.98	–	–	–
Day49	2.27	60	10	150	134	1.87	0.31	18.0	47.2	0.75	20.7	256,741.0	3576.75
Day51	2.07	66	10	–	–	–	–	19.6	50.3	1.10	–	–	–
Day53	1.95	57	6	143	126	1.54	0.21	18.8	57.2	1.53	–	356,495.0	2437.32
Day55	1.87	52	12	–	–	–	–	16.2	53.2	2.18	19.9	–	–

On day 41 the patient developed fever of 39.0°C, accompanied by fatigue and severe cough. The patient’s arterial partial pressure was: Pa_O2_ = 75 mm Hg, Pa_CO2_ = 42 mmHg, Sp_O2_ = 89%. By this time, the CART19 and CART22 DNA could be detectable in peripheral blood with a drastic increase. The absolute lymphocyte count was 2.9×10^9^/L and the CART19 and CART22 showed an increase on day 41 (**Figure 1D**). FCM analysis showed 5.38% CAR cells in the peripheral blood (**Figures 1E1**, **E2**). PB assessment revealed the normal CRP and PCT levels, indicating that the fever and respiratory symptoms were not caused by infection (**Table 1**). On day 43, the patient had the manifestations of hemophagocytic lymphohistiocytosis (HLH), characterized by the progressive descent in WBC, RBC, and PLT; enlarged spleen and liver; an increase in inflammatory cytokine-6(IL-6)and ferritin levels; and impaired coagulation function (**Table 1**). BM smear showed less than 0.5% immature lymphocytes and about 16% hemophagocytic cells in NEC (**Figure 1A3**).

Based on the clinical symptoms and the laboratory results, the patient was finally diagnosed with grade III CRS (12–14) and carHLH (15). On day 41, high-flow nasal cannula (8L/min), antibacterial treatment (mepitin and vancomycin), and tocilizumab were administered. The patient’s cough and hypoxemia resolved within 24 h (Pa_O2_ = 89 mm Hg, Pa_CO2_ = 39 mmHg, Sp_O2_ = 95%). However, fever was not controlled, therefore an anti-fungus drug (carpofennet) was administered after antibacterial treatment (mepitin and vancomycin) for 72h. On day 43, dexamethasone and etoposide were administered to the patient according to HLH-94 protocol (16). Unfortunately, she did not respond well to the treatment and eventually died of cerebral hemorrhage on day 55.

### Case 2

**Figure 2:**
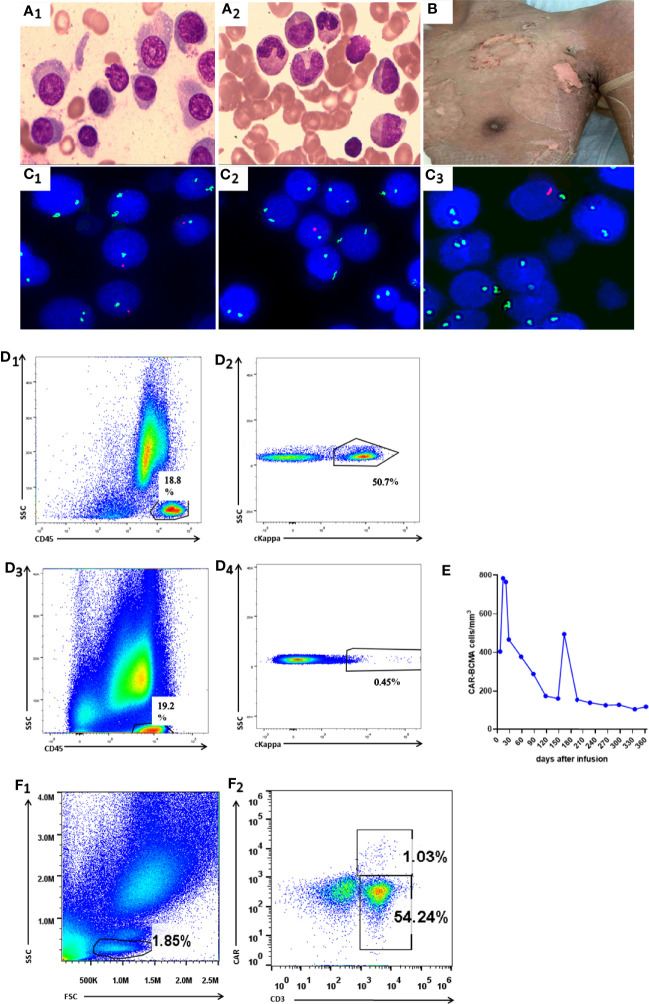
Manifestations in Case 2. **(A1)** BM smear before CART therapy showed that plasma cells accounted for 12.5% total cells. **(A2)** BM smear on day 90 showed that plasma cells accounted for 2.7% total cells. **(B)** The picture shows that skin rashes with blisters resolved on day 166 (3 days after methylprednisolone treatment). **(C1–C3)** FISH before CART therapy, on day 90 and on day 163 revealed XY[213]/XX[287], XY[16]/XX[484],and XY[6]/XX[494],respectively. **(D1, D2)** BM FCM analysis before CART therapy exhibited 50.9% sKappa+ cells. **(D3 D4)** BM FCM analysis on day 90 after CART therapy exhibited 0.45% sKappa+ cells. **(E)** FCM analysis showed CAR-BCMA cells in peripheral blood after CART therapy. **(F1, F2)** FCM analysis showed 1.03% CAR cells in the peripheral blood on day 163.

A male patient aged 38 years was diagnosed with plasmablastic lymphoma in August 2017. The FCM analysis of BM revealed that malignant cells accounted for approximately 6.2% of NEC and exhibited the expression pattern CD20^+^CD138^+^CD38^+^cIgG^+^cKappa^+^. After the first-line administration, he achieved CR and received HLA–matched sibling (sister) donor transplantation in February 2018. Cyclosporin A combined with methotrexate and mycophenolate mofetil were administered for graft-versus-host disease (GVHD) prophylaxis. On day 18 post-transplantation he obtained hematopoietic reconstitution and on day 30 he achieved complete donor chimerism. GVHD was not observed for 4 months after allo-HSCT. The patient was diagnosed with relapsed plasmablastic lymphoma in June 2018. The clinical features of relapse were as follows: 1) WBC, Hb, and Plt were 15.47 × 10^9^/L, 85 g/L, 45×10^9^/L, respectively; 2) the serum IgG ĸκ protein level was 21.6 g/L; 3) BM smear revealed that lymphoblast cells accounted for 12.5% of NEC (**Figure 2A1**) and FCM analysis showed that 50.3% of CD45^+^cells were characterized by cKappa (**Figures 2D1**, **D2**); 4) FISH showed XY[213]/XX[287] (**Figure 2C1**). Therefore, the patient was recruited into CART clinical trial for relapsed or refractory BCMA+ malignancies (ChiCTR-OPC-16009113). T cells for the construction of BCMA-CART were collected from the PB of HLA–matched sibling (sister) donor. Patients were given fludarabine 25 mg/m^2^ and cyclophosphamide 200 mg/kg on day -4, -3, and -2, followed by 6.2 × 10^6^/kg BCMA-CART on day 0 (17). On day 7, the peak of CART cells could be detected in the PB and the patient was diagnosed with grade I CRS, characterized by fever (38.2°C). His body temperature returned to normal after a dose of tocilizumab was administered. On day 30, the serum IgG κ protein level dropped to 8.05 g/L. On day 90, the serum IgGĸκ protein level was 4.31g/L. Lymphoblast cells accounted for 2.7% of NEC in BM smear (**Figure 2A2**). FCM analysis of BM showed 0.45% of CD45^+^cells were characterized by cKappa (**Figures 2D3**, **D4**) and FISH displayed XY[16]/XX[484] (**Figure 2C2**). These results indicated that the patient had achieved CR.

On day 163, the patient experienced fever (38.6°C), hypotension (88/52 mmHg), skin rashes, and large blisters all over the trunk. FISH showed 99.6% donor chimerism (XY[11]/XX[494]) in BM (**Figure 2C3**). The BCMA-CART showed a sharp elevation on day 163 (**Figure 2E**) and FCM analysis showed 1.03% CAR cells in the peripheral blood (**Figures 2F1**, **F2**). Moreover, the laboratory examinations indicating normal liver function, elevated IL-6, and ferritin levels are shown in **Table 2**.

**Table 2:** The laboratory results in Case 2.

Date After CART	WBC (10^9^/L)(Ref: 4-10)	Hb (g/L)(Ref: 110-150)	Plt (10^9^/L)(Ref: 150-390)	ALT (U/L)(Ref: 5-40)	AST (U/L)(Ref: 8-40)	CRP (mg/L)(Ref: 0-5)	PCT (μg/L)(Ref: 0-0.5)	Ferritin (ng/ml)(Ref: 15-200)	IL-6 (pg/ml)(Ref: 0.37-0.46)
Day163	10.16	103	98	9	18	14.32	0.15	508.4	3568.34
Day166	9.36	95	95	–	–	5.65	0.14	495.3	2453.86
Day171	6.19	85	97	35	28	3.42	0.16	432.6	385.67
Day176	8.02	92	102	–	–	4.01	0.20	74.1	40.90
Day181	7.81	101	95	–	–	–	–	–	–
Day186	9.30	107	104	–		–	–	53.2	22.38

Based on these results, the patient was diagnosed with grade III CRS (12–14). He was treated with dopamine, norepinephrine, tocilizumab, and methylprednisolone (1 mg/kg/day). Symptoms resolved within 8 h, and vasopressor support was discontinued. Within 3 days, patient’s condition gradually improved with the normalization of body temperature (36.7°C) and blood pressure (123/79 mmHg), unprogressive rashes (**Figure 2B**), as well as descending ferritin and IL-6 levels (**Table 2**). As the temperature stayed normal for 7 days, we stopped giving antimicrobial drugs to the patient on day 177. The dosage of methylprednisolone was reduced from 10 days after administration on day 173, and the drug was discontinued 3 weeks after reduction on day 194. His clinical response persisted for over 6.0 months.

## Discussion

The late adverse events occurring 30 days after CART therapy are defined as the delayed toxicity mainly manifested by cytopenia (18, 19). Severe CRS, including grade III and grade IV CRS, occurred within 22 days after the infusion (8, 9). In our study, case one and case two developed sCRS on day 41 and day 163, respectively. To the best of our knowledge, this is the first report of CART-cell inducing late sCRS.

In case one, the patient experienced sCRS and carHLH on an atypical onset time. On day 41, the patient was diagnosed with grade III CRS for a sudden high fever and hypoxemia with no evidence of HLH. On day 43, the patient had the manifestations of hemophagocytic lymphohistiocytosis (HLH), characterized by the progressive descent in WBC, RBC, and PLT; enlarged spleen and liver; and impaired coagulation function. Hence, the diagnosis of carHLH was established. CarHLH occurred in 26.7%-40.4% of patients who experienced CRS treated with CART therapy and the onset time ranged from 7 to 26 days post-CAR infusion (4, 20, 21). Clinical features of carHLH included hyperferritinemia, hypertriglyceridemia, hypofibrinogenemia, coagulopathy, hepatic transaminitis, hyperbilirubinemia, severe neutropenia, elevated lactate dehydrogenase, and occasionally hemophagocytosis. Furthermore, it suggests that the occurrence of CarHLH is related to the expansion and long-term existence of CART cells (20, 21). Consistent with the previous reports, hemophagocytic cells were found in the bone marrow smear and CART expansion was observed. The relationship between CRS and carHLH is still inconclusive. Some studies believe that carHLH is a variant of CRS, and it is not recommended to incorporate HLH into CRS for diagnosis and treatment (20, 21). CRS was considered an immune trigger leading to HLH and was a part of a spectrum of systemic hyper-inflammatory disorders (22). It was reported that CRS can be successfully ameliorated with the IL6R inhibitor tocilizumab and does not appear to decrease efficacy of the CART cell (23, 24). Unfortunately, she was not responsive to tocilizumab and HLH-94 protocol, consequently dying of HLH progression.

In case two, the patient received BCMA-CART therapy 4 months after allo-HSCT. On day 163 after CART, the patient suffered from fever, hypotension, and skin lesions. Based on clinical manifestations and laboratory assessments, the diagnosis of grade III CRS was established. Cutaneous toxic effects are reported as a kind of toxic reaction after CART treatment. Rubin et al. described four patients with skin lesions attributed to CAR therapy, including two patients with eruptions with unusual mononuclear cell dermal infiltrates and two patients with transient eruptions (25). Moreover, Yongxian Hu et al. has reported a patient receiving CD19/CD22 dual-targeted CART therapy experienced cutaneous toxic effects and grade III CRS. The existence of predominant CART cells in the bullae fluid and the huge discrepancy in cytokine levels proves that bullae fluid cytokines were produced *in situ* by infiltrated CART cells (26). After two doses of tocilizumab were administered, the cutaneous toxic effects including the fingertip cyanosis, swelling, and healing of the cutaneous lesions was resolved (26). These findings suggest the development of a local toxic reaction with cutaneous involvement. Consistent with previous reports, our case was considered as sCRS rather than acute GVHD for the normal liver function, higher IL-6 levels, and the drug response. Firstly, the target organs in patients with GVHD include the intestinal tract, skin, liver, and kidney. Despite the presence of skin lesions, liver function in this patient remained normal. Secondly, in case two IL-6 levels elevated up to 3568.34pg/ml, while it was reported that in recipients with acute GVHD IL-6 levels were 10-40pg/ml (27). Thirdly, the patient had a rapid response to tocilizumab, suggesting that the elevated cytokines might be a cause of cutaneous toxic effects.

CART re-expansion might be the cause of the late sCRS. Driven by the supraphysiologic secretion of proinflammatory cytokines, CRS is remarkably associated with active T cells and tumor burden (28, 29). CART served as the main reason for sCRS, because two patients remained CR and a sharp increase of CART in the PB was found. CART has been reported to exist for 23.1 months in peripheral blood (11, 17, 30). The expansion of CART cells is linked with the structural composition of the CAR (e.g., single-chain spacer, extracellular, and costimulatory domains) and the *in vivo* environment of the recipient (e.g., elevated cytokines, CD4:CD8 T cell ratio and frequency) (31–34). The underlying factors in two cases might trigger the CART re-expansion. In case one, the patient received a sequential infusion approach with CART19 and CART22. On day 7, CART19 took a higher proportion in peripheral blood than CART22 (3475.0 *vs*. 2345.0 copies/μg of genomic DNA) while on day 41 CART22 was predominant (14569.0 *vs*. 6952.0 copies/μg of genomic DNA). Patients with CART22 and CART19 developed CRS at different times (21). Whether the proportionate shift resulted in the re-expansion of CART cells needed to be further clarified. In case two, on day 163 the laboratory examination showed elevated CRP (**Table 2**), suggesting that inflammatory cascade may be the reason for CART re-expansion. Elevation of cytokines, such as IL-15, IL-21, IL-6, and IL-7, has been reported to promote the expansion of CART (35–37). We speculated that due to different factors cytokines partially increased, resulting in the expansion of CART.

In conclusion, severe CRS could occur in the late period after CART therapy and re-expansion of CART cells possessed the potential risk for late sCRS. The data presented are limited due to the low number of patients and retrospective nature of the study. Besides, further investigations into the cytokine profiles (IL-1β, IL-8, IL-4) and the treatment of sCRS (anakinra) might improve the sensitivity of the diagnosis and the effectiveness of treatment. It nevertheless highlights the necessity for concentrating on proliferation and activation of CART inducing sCRS in the clinical work.

## Patient Perspective

The patients provided their written informed consent to participate in this study. Written informed consent was obtained from the individual(s) for the publication of any potentially identifiable images or data included in this article.

## Data Availability Statement

The raw data supporting the conclusions of this article will be made available by the authors, without undue reservation.

## Ethics Statement

Ethical review and approval were not required for the study on human participants in accordance with the local legislation and institutional requirements. The patients provided their written informed consent to participate in this study. Written informed consent was obtained from the individual(s) for the publication of any potentially identifiable images or data included in this article.

## Author Contributions

QL and PL conceived and designed the study. JH, NX, and HZ provided the study materials or patients. YZ, RC, JX, and TL performed the experiments. JH, DW, and RZ wrote the manuscript. All authors contributed to the article and approved the submitted version.

## Funding

This study was supported by National Key Research and Development Programme of China (No.2017YFA105500,No.2017YFA105504), National Natural Science Foundation of China(No.81770190,No.81970161), Research and Development Program in Key Areas of Guangdong Province (No.2019B020236004),Natural Science Foundation of Guangdong Province (No.2019A1515011924).

## Conflict of Interest

The authors declare that the research was conducted in the absence of any commercial or financial relationships that could be construed as a potential conflict of interest.

## Publisher’s Note

All claims expressed in this article are solely those of the authors and do not necessarily represent those of their affiliated organizations, or those of the publisher, the editors and the reviewers. Any product that may be evaluated in this article, or claim that may be made by its manufacturer, is not guaranteed or endorsed by the publisher.
