# Supplementary material for: Case Report: Chimeric Antigen Receptor T Cells Induced Late Severe Cytokine Release Syndrome
Source: Front Oncol. 2022 Jun 1;12:893928. doi: 10.3389/fonc.2022.893928 (PMC9198280; doi:10.3389/fonc.2022.893928)
Supplement: Supplementary file 1 [file DataSheet_1.docx]

**Supplementary Materials**

**Generation and expansion of CART**

Validation of the CAR constructs and procedures for cell production and quality control assays were conducted according to previous reports(1, 2). The CART19 and CART22 were composed of a single-chain variable fragment derived from a murine monoclonal antibody against human CD19 or CD22, 2 costimulatory domains from CD28 and 4-1BB, and the CD3-ζ chain as the activation domain. The BCMA-CART contained a single-chain variable fragment from a murine monoclonal antibody against human BCMA, a costimulatory domain from CD28, as well as the CD3-ζ chain as the activation domain.

**CART detection**

As the the previous reports described(1, 2), CART19 and CART22 in blood were measured by quantitative PCR and flow cytometry, and BCMA-CART were detected by flow cytometry. Detection after CART administration were performed weekly in the first month and monthly in the first year .

**Toxicity Evaluations**

CRS was prospectively graded using the Penn criteria(3, 4), and reconciled with ASTCT CRS consensus grading system(5). HLH was assessed by HLH-2004 criteria(6).

**References:**

1.Wang N, Hu X, Cao W, Li C, Xiao Y, Cao Y, Gu C, Zhang S, Chen L, Cheng J, Wang G, Zhou X, Zheng M, Mao X, Jiang L, Wang D, Wang Q, Lou Y, Cai H, Yan D, Zhang Y, Zhang T, Zhou J, Huang L. Efficacy and safety of CAR19/22 T-cell cocktail therapy in patients with refractory/relapsed B-cell malignancies. *BLOOD*.(2020)135: 17-27. doi:10.1182/blood.2019000017

2.Xu J, Wang Q, Xu H, Gu C, Jiang L, Wang J, Wang D, Xu B, Mao X, Wang J, Wang Z, Xiao Y, Zhang Y, Li C, Zhou J. Anti-BCMA CAR-T cells for treatment of plasma cell dyscrasia: case report on POEMS syndrome and multiple myeloma. *J HEMATOL ONCOL*.(2018)11: 128. doi:10.1186/s13045-018-0672-7

3.Porter D, Frey N, Wood PA, Weng Y, Grupp SA. Grading of cytokine release syndrome associated with the CAR T cell therapy tisagenlecleucel. *J HEMATOL ONCOL*.(2018)11: 35. doi:10.1186/s13045-018-0571-y

4.Porter D, Frey N, Wood PA, Weng Y, Grupp SA. Correction to: Grading of cytokine release syndrome associated with the CAR T cell therapy tisagenlecleucel. *J HEMATOL ONCOL*.(2018)11: 81. doi:10.1186/s13045-018-0627-z

5.Lee DW, Santomasso BD, Locke FL, Ghobadi A, Turtle CJ, Brudno JN, Maus MV, Park JH, Mead E, Pavletic S, Go WY, Eldjerou L, Gardner RA, Frey N, Curran KJ, Peggs K, Pasquini M, DiPersio JF, van den Brink M, Komanduri KV, Grupp SA, Neelapu SS. ASTCT Consensus Grading for Cytokine Release Syndrome and Neurologic Toxicity Associated with Immune Effector Cells. *Biol Blood Marrow Transplant*.(2019)25: 625-638. doi:10.1016/j.bbmt.2018.12.758

6.Henter JI, Horne A, Arico M, Egeler RM, Filipovich AH, Imashuku S, Ladisch S, McClain K, Webb D, Winiarski J, Janka G. HLH-2004: Diagnostic and therapeutic guidelines for hemophagocytic lymphohistiocytosis. *PEDIATR BLOOD CANCER*.(2007)48: 124-31. doi:10.1002/pbc.21039
